# Application of molecular source tracking and mass balance approach to identify potential sources of fecal indicator bacteria in a tropical river

**DOI:** 10.1371/journal.pone.0232054

**Published:** 2020-04-30

**Authors:** Kevan M. Yamahara, Daniel P. Keymer, Blythe A. Layton, Sarah P. Walters, Rachelle S. Thompson, Matt Rosener, Alexandria B. Boehm

**Affiliations:** 1 Department of Civil & Environmental Engineering, Stanford University, Stanford, CA, United States of America; 2 Waipā Foundation, Hanalei, Kaua'i, Hawai'i, United States of America; University of Notre Dame, UNITED STATES

## Abstract

Microbial source tracking and a mass balance approach were used to identify sources of fecal indicator bacteria (FIB) in the Hanalei River, Kaua’i, Hawai’i. Historically, concentrations enterococci and *Clostridium perfringens* were significantly higher during storm flows compared to non-storm flows in the Hanalei River, and correlated to total suspended solids in the river. During targeted dry weather studies, the Hanalei River bed sediments and streambank soils were documented to harbor *E*. *coli*, enterococci, and the human- and pig-specific fecal markers in *Bacteroidales*, suggesting that sediments and soils may be potential sources of these microorganisms to the Hanalei river. The human-specific marker in *Bacteroidales* was four times as likely to be detected in sediment and soil samples as in water samples. Furthermore, the occurrence of host-specific source tracking markers is indicative that a portion of FIB present in the Hanalei River are of fecal origin. A mass balance approach was used to explore causes of observed FIB loadings and losses along different reaches of the river. Resuspension or deposition of FIB-laden river sediments cannot account for changes in *E*. *coli* and enterococci concentrations along the river during dry weather. Additionally, losses due to bacterial inactivation were insignificant. Groundwater and ditches draining agricultural and urban lands were shown to provide sufficient FIB fluxes to account for the observed loads along some river reaches. The presence of the human-specific *Bacteroidales* marker in the river water, sediments and adjacent soils, as well as the presence of the human enterovirus marker in the water, suggests that there is widespread human fecal contamination in the Hanalei River that is likely a result of nearby wastewater disposal systems.

## Introduction

Elevated levels of fecal indicator bacteria (FIB, including *Escherichia coli* and enterococci) impair beneficial uses of surface waters. In order to reduce high FIB levels in surface waters, US Clean Water Act Total Maximum Daily Loads (TMDLs) and EU Water Framework Directive Programmes of Measures are established and require pollution control measures to be implemented. Both aim to reduce source loadings and help achieve acceptable water quality. FIB are the number one cause of impairment for US surface waters (14.3% are impaired [1]), so a need exists to better understand their sources and fate in the environment.

The apportionment of microbial fluxes from different sources is challenging because of the myriad of potential sources of pollution that can lead to elevated FIB levels. FIB can be found in a number of fecal [2–5] and non-fecal (e.g., in soil and on plants) [6,7] sources, many of which are non-point in nature. The ability to distinguish between environmental sources (e.g. sediments), which may be impossible to control, and anthropogenic sources, which are controllable, is essential to develop successful plans for pollutant load reductions. Additionally, distinguishing fecal from non-fecal sources of FIB may be important from a health risk perspective, since non-fecal FIB may not be associated with increased health risks [8]. In tropical regions like Hawaii, the problem of FIB source identification is exacerbated by the fact that these organisms may be indigenous to tropical soils and waters [9–11]. For this reason, the Department of Health in Hawaii uses *Clostridum perfringens*, in addition to enterococci, as an indicator to assess water quality.

Microbial source tracking is an approach for identifying sources of fecal indicator bacteria. *Bacteroidales* markers are popular DNA-based microbial fecal source tracking tools. They have been used extensively in studies of temperate and subtropical regions [12–15]. However, there has only been limited work using *Bacteroidales* in the tropics [16,17]. Host-specific markers in *Bacteroidales* are more specific than other microbial source tracking tools [18–20]. *Bacteroidales* DNA markers have been developed for human [21], ruminant [22], swine [23], dog [24], and horse [23] feces. The bacteria that contain these source-specific DNA markers have not been cultivated, so the markers can only be measured using culture-independent methods such as polymerase chain reaction (PCR). Human viruses have also been used as microbial source tracking targets; the presence of human viruses in coastal waters is both strong evidence of a human contamination source and a health risk. Enteroviruses, which are single-stranded, plus sense, RNA viruses, have been used to assist in microbial source tracking throughout the developed world [12,25,26].

**Fig 1:**
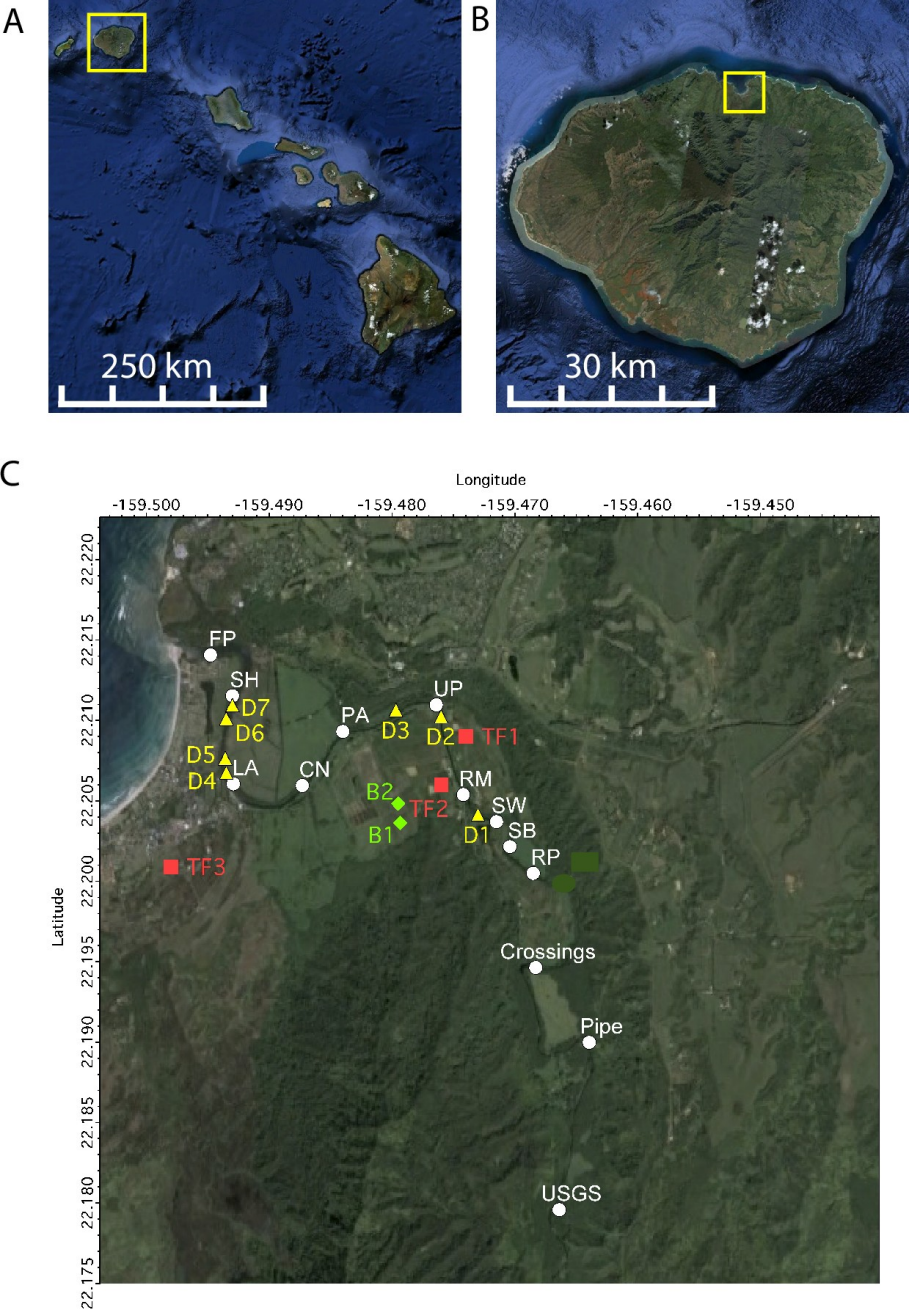
Map of study site. A. Hawaiian Archipelago, with the island of Kaua`i boxed in yellow. B. Island of Kaua`i. The yellow rectangle indicates the study area. C. Study area. White circles denote river cruise sites, yellow triangles denote locations of ditch samples, red boxes denote taro lo'i field (TF) samples, and green diamonds represent bird refuge pond (B) samples.

The present study investigates the sources and loading of FIB to a tropical river draining a rural watershed, the Hanalei River, Kaua’i, Hawai’i (Fig 1). A mass-balance approach is complemented by molecular source tracking markers to identify likely sources of FIB to the river. Specifically, the role of soils and sediments, as well as drainage ditches and groundwater as sources is investigated. The Hanalei River was chosen for this study because routine water quality monitoring by the Hawaii Department of Health has shown that beaches adjacent to the Hanalei River have poor microbial water quality.

There are numerous potential point and non-point sources of microbial pollutants along the Hanalei River. The Hanalei River drains a 61.2 km^2^ rural watershed that is primarily forested (95.2%) with 2.3% of the land cover cultivated and 0.8% urban [17]. Most cultivated and urban land covers are located directly adjacent to the river reach closest to the ocean (Fig 1). All residences utilize on-site wastewater disposal, including both cesspools and septic systems, so groundwater discharge to the river is a potential source of bacterial pollution. Taro, the most common crop, is cultivated in flooded fields, or lo`i, where a constant flow of water is applied to the crop and then discharged into the Hanalei River via return ditches. Lo`i are typically fertilized and attract waterfowl, so they could represent microbial pollutant sources. Domesticated animals such as cows, goats, pigs, horses, and chickens are kept in enclosures adjacent to some of the cultivated lands, and feral pigs and goats roam forested portions of the watersheds. In the lowest reaches of the river, adjacent to Hanalei town, there are several ditches that drain a combination of agricultural and urban runoff into the Hanalei River; waterfowl and other birds are common throughout the lower portion of the watershed. Finally, tropical soils and sediments are documented reservoirs of FIB [6] and represent sources of FIB to adjacent surface waters.

**Fig 2:**
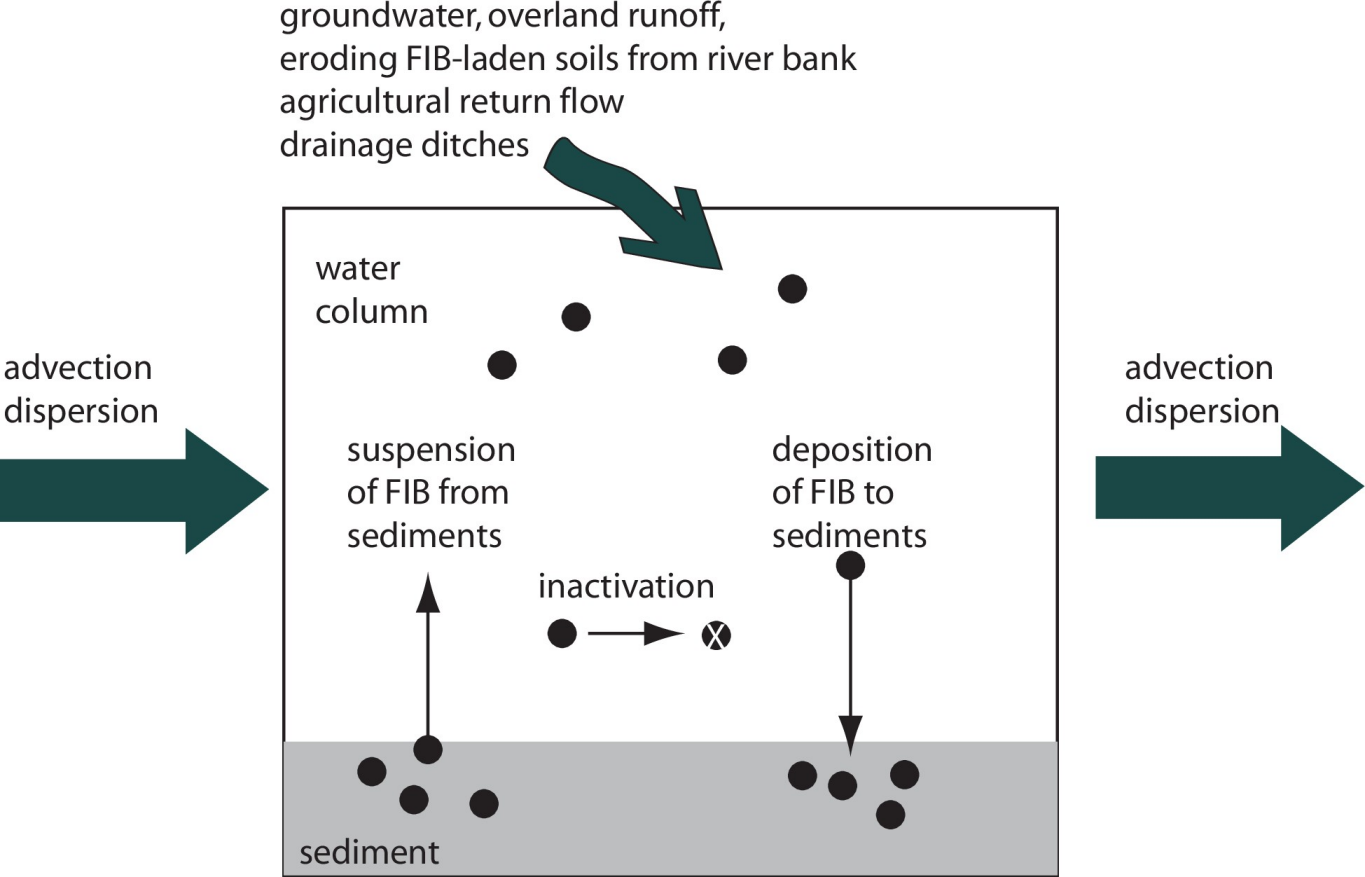
Conceptual model illustrating the endogenous and exogenous inputs and losses of FIB to a water parcel in the Hanalei River.

A generalized conceptual model illustrating pollutant sources and sinks within a segment of the river is shown in Fig 2. Exogenous inputs to the river include polluted groundwater, overland flow during storm events, soils resulting from erosion of land including river banks, and discharges from agricultural and urban land covers (e.g., ditches). Endogenous inputs include suspension of contaminated river sediments. Pollutant sinks include deposition to the sediments as well as inactivation. During storm conditions, all of these sources may be active. However, during dry conditions, inputs due to erosion of soils and overland flow are expected to be minimal.

## Materials and methods

### Site description

No permit was needed for the field research. Two field studies, in March 2008 and March 2009, were conducted within the Hanalei River watershed on the rural north shore of the island of Kaua’i, Hawaii (Fig 1). The Hanalei River, the only American Heritage River in the State of Hawai’i, flows 26 km beginning on the eastern slopes Mt. Wai`ale`ale and discharging to the Pacific Ocean at Hanalei Bay. The average yearly flow rates in the Hanalei River during 2008 and 2009 were 5.4 m^3^/s and 6.2 m^3^/s, respectively. River depths and widths during these studies ranged from 0.6 m to 2.1 m deep and 4.5 m to 24.3 m wide.

### Historical data

Concentrations of enterococci (ENT) and *Clostridium perfringens* (CP) in water samples collected between July 1999 and November 2009 from a location approximately 230 m upstream of the Hanalei River mouth (22°12'49.01" N, 159°29'45.21" W, Fig 1) were obtained from the Hawaii State Department of Health. Daily average volumetric flow rates and total suspended solids (TSS) concentrations in the river were obtained from a USGS gauging station located approximately 8200 m upstream of the river mouth (http://waterdata.usgs.gov/, Fig 1, site 16103000, 22°10'46.5" N, 159°27'59.0" W). To characterize the rainfall during and prior to the study, daily rainfall was obtained from the USGS (http://waterdata.usgs.gov, site 221101159280801). Data were compiled through 2009 as this is the latest year that field data presented here were collected (see below).

### River cruises

On 29 and 30 March 2008 and 15 and 19 March 2009, sampling was conducted along the length of the Hanalei River to obtain along-river spatial distributions of TSS in water and FIB, host-specific *Bacteroidales*, and enterovirus source tracking markers in the water, sediments and river bank soils. These four sampling events are referred to as river cruise (RC) 1, RC2, RC3, and RC4, respectively. Each river cruise commenced at 0500 h and finished by 0700 h local time, thus eliminating any effects of microbial photoinactivation [27]. A total of 13 locations were sampled along the length of the river, 10 by kayak and 3 by foot (Fig 1, Table 1). Sampling conducted by kayak began at a site located upwatershed of most of the cultivated and urban land cover (site RP, located 6135 river meters from the outlet of the river) and continued at approximately 550 m intervals, as the river flowed through the developed portion of the watershed (Fig 1). Additional samples were collected by foot at the USGS gauge station located 8240 m from the river mouth (USGS), at a dirt road crossing located 6950 m from the river mouth (Crossings) and at a river intake pipe located 7600 m from the river mouth (Pipe) Pipe was sampled on 26 and 29 March 2008 and 17 and 19 March 2009, and these samples were incorporated into RC1, RC2, RC3 and RC4, respectively. Crossings was sampled on 26 March 2008 and 17 March 2009, and this data was used in the RC1 and RC3 data, respectively. The USGS sampling site was sampled on 17 March 2009 and was only utilized in the RC3 data.

**Table 1:** Study sampling locations. Sampling sites denoted by sample type and corresponding GPS coordinates and sediment types. Distances (meters) from the USGS gauge is provided next to the sample type.

Sampling Site	Sample Type	Latitude	Longitude	Sediment Type
USGS Gauge	River Cruise (0)	N 22°10’46.56”	W 159°27’57.6”	Gravel
Pipe	River Cruise (1300)	N 22°11’24”	W 159°27’50.4”	N/A
Crossings	River Cruise (2400)	N 22°11’40.56”	W 159°28’4.8”	Sand
RP	River Cruise (3100)	N 22°12’1.8”	W 159°28’8.4”	Silt
SB	River Cruise (3360)	N 22°12’7.92”	W 159°28’12”	Gravel
SW	River Cruise (3810)	N 22°12’13.32”	W 159°28’19.2”	Gravel
RM	River Cruise (4230)	N 22°12’19.44”	W 159°28’26.4”	Gravel
UP	River Cruise (4900)	N 22°12’39.6”	W 159°28’33.6”	Sand
PA	River Cruise (5425)	N 22°12’33.48”	W 159°29’2.4”	Sand
CN	River Cruise (6135)	N 22°12’21.6”	W 159°29’13.2”	Sand
LA	River Cruise (6950)	N 22°12’21.96”	W 159°29’34.8”	Sand
SH	River Cruise (7600)	N 22°12’41.4”	W 159°29’34.8”	Sand
FP	River Cruise (8240)	N 22°12’50.76”	W 159°29’42”	Silt
D1	Ditch (4020)	N 22°12’14.4”	W 159°28’22.8”	Silt
D2	Ditch (4800)	N 22°12’37.8”	W 159°28’48”	Silt
D3	Ditch (5150)	N 22°12’36.36”	W 159°28’33.6”	Silt
D4	Ditch (7000)	N 22°12’23.76”	W 159°29’38.4”	Silt
D5	Ditch (7100)	N 22°12’27”	W 159°29’38.4”	Silt
D6	Ditch (7300)	N 22°12’36”	W 159°29’38.4”	Silt
D7	Ditch (7500)	N 22°12’38.88”	W 159°29’34.8”	Silt
TF1	Taro Field	N 22°12’32.4”	W 159°28’26.4”	Silt
TF2	Taro Field	N 22°12’21.6”	W 159°28’33.6”	Silt
TF3	Taro Field	N 22°12’3.24”	W 159°29’52.8”	Silt
B1	Bird Pond	N 22°12’12.96”	W 159°28’44.4”	Silt
B2	Bird Pond	N 22°12’17.28”	W 159°28’48”	Silt

At each site, 1-L water samples were collected from mid-river at a depth of 30 cm below the water's surface in sterile 10% HCl-acid washed polypropylene bottles. It was assumed that these samples were representative of the entire water column. River sediments from the top 2.5 cm of the river bed were collected in sterile 50 mL polypropylene centrifuge tubes by a diver. During the 2009 cruises, river bank soil samples were collected from above the water line to a depth of 2.5 cm at each sampling site using sterile 50 mL polypropylene centrifuge tubes. A total of 47 water, 45 sediment, and 22 soil samples were collected during river cruises. Water, sediment and soil samples were tested for *E*. *coli* (EC), enterococci (ENT), enteroviruses (EV) and the ruminant-, pig-, and human-specific fecal markers in *Bacteroidales* (CF, PF and HF markers, respectively). However, enteroviruses in sediments were only analyzed during 2009. Particle diameter (d_p_) was used to classify sediments, based on the majority of the grain size present, as gravel d_p_ > 0.5 mm), sand (0.05 mm > d_p_ > 0.5 mm) or silt (d_p_ < 0.05 mm).

### Potential sources

Ditches flowing into the Hanalei River were sampled in both 2008 and 2009 (Fig 1, Table 1). In 2008, water samples were collected from 3 ditches discharging into the Hanalei River. A sample from ditch 1 (D1) was collected during RC1, and samples from ditches 2 and 3 (D2 and D3, respectively) were collected by foot immediately following RC1. In 2009, water and sediment samples were collected from the same ditches during RC4. On 18 March 2009, water and sediment samples were collected from four ditches (D4, D5, D6, and D7) (Fig 1). Water and sediment samples were collected from 3 taro lo'i and 2 bird refuge ponds in March 2009 (TF1, TF2, and TF3, and B1 and B2) (Table 1). All samples were collected as previously described (above) and assayed for EC and ENT concentrations as well as EV, CF, PF and HF source tracking markers.

### Analytical methods

Enterococci (ENT) and *E*. *coli* (EC) concentrations were measured using IDEXX defined substrate assays (Colilert-18 and Enterolert, IDEXX, Westbrook, ME) within 6 h of sample collection. ENT and EC were eluted from sediment and soil samples using sterile, distilled water as previously described for sands [28], with a modified settling time of 5 min. Ten milliliters of water or eluants were added to 90 mL of Butterfield's Buffer (Weber Scientific, Hamilton, NJ) containing the appropriate IDEXX defined substrate for enumeration in Quantitray 2000 (IDEXX). The dry weights of sediments and soils, used for the standardization of microbial concentrations, were determined by weighing sediments/soils after heating at 110°C for 24 hr. All bacterial concentrations in sediments and soils are reported by dry weight. The waterborne concentrations in the river are the total water column concentration and has contributions from both suspended sediments and planktonic organisms. The sediment and soil concentrations represent the total bacteria present in the sample, which sometimes included some porewater. It was not possible to determine what, if any, fraction of the sediment/soil FIB were present in porewater as opposed to sediment. We assume that FIB measured in sediments and soils are primarily those associated with sediments and porewaters contribute little FIB to the measurement. The lower and upper detection limits for ENT and EC in water samples are 10 and 24192 Most Probable Number (MPN)/100 mL, respectively. Lower and upper detection limits for ENT and EC in sediment and soil samples vary slightly due to differences in dry weight assayed, but are approximately 0.2 and 9837 MPN/ g dry weight, respectively.

Turbidity was measured in all water samples using a bench top turbidimeter (Model DRT-15CE, HF Scientific, Ft. Meyers, FL). To calculate TSS from the turbidity data, nephelometric turbidity units (NTU) were converted to mg/L using a calibration curve generated for TSS plotted against NTU for the Hanalei River: TSS (mg/L) = 1.1 (NTU) [29].

Samples were analyzed for human-specific, ruminant-specific, and pig-specific fecal *Bacteroidales* markers (HF, CF, and PF markers, respectively) and enterovirus (EV) using conventional PCR and RT-PCR. Although quantitative versions of these assays exist, we opted for convention versions due limited resources available for the field work. In samples collected during 2008, 100 mL of each water sample were filtered through a 0.45 μm pore size Supor filter (Millipore, Billerica, MA), and 500 mL were filtered through a 0.45 μm pore size HA filter (Millipore, Billerica, MA) for bacterial and viral analyses, respectively [30–32]. During 2009, both bacterial and viral fractions were co-concentrated by filtering 500 mL of each water sample through a 0.45 μm pore size HA filter (Millipore, Billerica, MA) [32]. Filters were stored in 2 oz Whirlpak bags (Nasco, Fort Atkinson, WI) at -80°C until analysis. For sediments and soils, 5 to 10 mL of eluant were filtered as described previously and stored similarly to water samples.

DNA was extracted from Supor filters using a modified DNeasy Tissue Kit (Qiagen, Valencia, CA) protocol [30]. Briefly, 500 μL of guanidine isothiocyanate (GITC) buffer (5 M guanidine thiocyanate, 100 mM EDTA, 0.5% N-lauroyl sarcosine; Sigma–Aldrich) were added to each Whirlpak bag. Filters were massaged by hand for 1 min and 500 μL of buffer AL was added to the filter and GITC. Lysates were then transferred to a clean 1.5 mL microcentrifuge tube and 500 μL of 100% ethanol was added to each sample. From this point, lysates were processed using manufacturer's protocols and purified DNA was eluted in 50 μL EB buffer.

DNA and RNA were extracted and purified from HA filters using modifications of the AllPrep DNA/RNA Micro Kit (Qiagen, Valencia, CA) [33]. One milliliter of RLT plus buffer with 10 μL β-mercaptoethanol and 20 ng of carrier RNA (Qiagen, Valencia, CA) was added directly to each Whirlpak bag with a filter and allowed to soak for 10 min. Lysates were pipetted from Whirlpak bags into 2 mL microcentrifuge tubes, and 1 mL of 70% ethanol was added to the lysate. Lysates were applied to an AllPrep DNA spin column and were processed from this point forward using manufacturer's protocols. Purified DNA was eluted in 50 μL EB buffer, and purified RNA was eluted in 14 μL RNase free water. DNA and RNA extracts were stored at -20°C and -80°C, respectively. In order to have consistent detection limits between years when different sample volumes were filtered, the DNA extracts from 2009 were diluted 1:5 in DNAse/RNase free water.

HF and CF marker PCR reactions consisted of the following: 2 μL of DNA extract, 1X Takara ExTaq PCR Buffer, 200 μM Takara ExTaq dNTPs, 0.2 μM HF183 or CF193 forward primer (Table 2), 0.2 μM Bac708 reverse primer (Table 2), 0.08% bovine serum albumin fraction V (GIBCO, Carlsbad, CA) and 0.025 Units/μL Takara ExTaq. The PF reaction consisted of the following: 2 μL of DNA template, 1X PCR Gold Buffer (Applied Biosystems, Foster City, CA), 1.5 mM PCR Gold MgCl_2_ 200 μM Takara ExTaq dNTPs, 0.2 μM PF163 forward primer, 0.2 μM Bac708 reverse primer (Table 2), 0.08% bovine serum albumin fraction V (GIBCO, Carlsbad, CA) and 0.025 Units/μL Takara ExTaq. PCR reactions were performed on a ABI 9700 GeneAmp PCR (Applied Biosystems, Foster City, CA) system using the following thermal cycling conditions; initial denaturation at 95°C for 2 min, 35 cycles of 45 s of denaturation at 95°C, 45 s of annealing at specified anneal temperatures (see Table 2), 45 s of extension at 72°C, and a final extension of 7 min at 72°C. Visualization of all amplified DNA products was performed by electrophoresis in 1.5% agarose gels stained with 0.5 μg/mL ethidium bromide. Positive PCR reactions produced DNA fragments of 525 bp (HF), 515 bp (CF), and 545 bp (PF). The detection limit for the PCR amplification was approximately 40 copies of target per reaction. This was determined by diluting a standard and testing it by PCR, and determining the lowest standard at which a band in the gel could be visualized. Given the volume of template run in each reaction, this corresponds to a detection limit of 200 copies per 100 mL water and 240 copies per g dry sediment or soil.

**Table 2:** Primer sequences for *Bacteroidales* host-specific fecal markers and enterovirus PCR and RT-PCR assays.

Primer ID	Sequence (5’ - 3’)	Size (bp)	Annealing Temp.(° C)	Reference
BAC708R	CAATCGGAGTTCTTCGTG	-	-	[21]
HF183F	ATCATGAGTTCACATGTCCG	525	61	[21]
CF193F	TATGAAAGCTCCGGCC	515	61	[21]
PF163	GCGGATTAATACCGTATGA	545	61	[23]
EVupstream	CCTCCGGCCCCTGAATG	-	-	[25]
EVdownstream	ACCGGATGGCCAATCCAA	196	59	[25]

“-”is provided in the size and annealing temperature of the reverse primers.

Enteroviruses were detected using reverse-transcriptase PCR (RT-PCR) in 25 μL reaction volumes with the following composition: 5 μL RNA template, 1X Qiagen One-Step RT-PCR Buffer, 400 μM Qiagen dNTP mix, 0.6 μM EV upstream primer, 0.6 μM EV downstream primer and 1X Qiagen One-Step RT-PCR Enzyme mix. PCR reactions were performed on a ABI 9700 GeneAmp PCR system using the following thermal cycling conditions: reverse transcription step at 50°C for 30 min and an initial PCR activation step at 95°C for 15 min, followed by 40 cycles of 30 s at 95°C, 30 s at 59°C, 1 min at 72°C, and a final extension of 10 min at 72°C. Positive RT-PCR reactions produced DNA fragments of 196 bp. The detection limit of the RT-PCR is approximately 40 copies or 56 copies per 100 mL water and 66 copies per 100 g dry sediment or soil.

### Mass balance approach for interpreting waterborne fecal indicator bacteria and total suspended solids

Observed loadings, L_obs_ (MPN/s) of EC and ENT were estimated for the 12 individual river segments studied during the river cruise. The river segments are reaches of the river defined by the 13 sample sites (Fig 1). Loadings were calculated for each year by combining measurements of FIB obtained during the RCs from 2008 (RC1 and RC2) and 2009 (RC3 and RC4), respectively. Water column and sediment FIB concentrations at each sampling site from RC1 and RC2 and from RC3 and RC4 were composited by combining the MPN results from the QuantiTrays. The total number of large and small wells from trays measuring FIB in water or sediment from the same site during the two relevant river cruises were summed together and MPN equations reported by Hurley and Roscoe [34] were used to estimate composite concentrations (MPN/100 mL) and their standard deviations.

The observed loading, L_obs_, in a river segment was calculated as the difference between the measured flux in and out of the river segment as follows:

$$L_{obs}=Q\left(C_{out}‑C_{in}\right)$$ (1)

where Q is the volumetric flow rate (m^3^/s), and C_in_ and C_out_ are the upstream and downstream measured concentrations of FIB (MPN/m^3^), respectively. Standard deviations of the concentrations were propagated to estimate the error for each observed load, L_obs_.

A schematic of possible sources and sinks of FIB along the river are shown in Fig 2. To assess whether resuspension and deposition of sediment from the river-bed could account for the changes in FIB load in the various river segments, we calculated the load of TSS in each river segment, and multiplied the TSS load by the concentration of FIB in the sediments measured within that river segment. This requires assuming that resuspended sediments have the same concentration of FIB as measured in the river-bed sediments, and that deposited TSS has the same concentration as those measured in the sediments. TSS load was calculated using a modified version of Eq (1) where C is concentration of total suspended solids.

We subsequently investigated the contribution of inactivation to FIB loading within the river segments. The differential equation representing change of FIB within a river segment due to inactivation is:

$$\frac{\partial C}{\partial t}=-u\frac{\partial C}{\partial x}-kC$$ (2)

where t is time, x is the position along the river, u is the river velocity (m/s), C(x) is the concentration of FIB in the water at a distance x (m) along the length of the river (MPN/100 mL)- this measurement includes contributions from planktonic and sediment-associated suspended bacteria, and k is the first order FIB inactivation rate constant (1/s) which is assumed to be first order with respect to C. Published dark inactivation rates of 3.3 x 10^−6^ s^-1^ for ENT [35] and 6.4 x 10^−6^ s^-1^ for EC [36] were used as inactivation rate constants within the model. The solution to Eq 2, assuming steady state conditions, is *C*(*θ*) = *C_o_e^−kθ^* where θ = x/u where u is the velocity in the river segment and Co is the concentration at the upstream end of the segment and C is the concentration at the downstream end. A steady state assumption was deemed appropriate based on the lack of rainfall 5 days prior to and during the field study and the steady flow conditions encountered during the river cruises. We calculated the change in load attributable to inactivation as Q(C- Co).

To determine whether microbial loadings from ditches could account for the observed FIB load within river segments where ditch discharges were located, the volumetric flow rate of the ditch, Q_dit_ (m^3^/s), that would be needed to obtain the observed load L_obs_, was calculated using the following equation:

$$Q_{dit}=L_{obs}/C_{dit}$$ (3)

where C_dit_ is the average concentration (MPN/m^3^) for that ditch measured in the appropriate year. This approach was necessary as flow rates from ditches were unmeasurable during this study.

Groundwater was also investigated as a possible source of FIB to the river. The required loading from a groundwater source, S (MPN per time per length of river in meters), to account for the observed loadings L_obs_ was estimated using the following equation:

$$S=L_{obs}/x$$ (4)

where and x is the length of the river segment (m). This formulation assumes that groundwater is the only source of FIB and does not consider additional sources such as ditches that may also be present in the river segments.

### Statistical analyses

Baseflow for the Hanalei River was determined by the local minimum method [37,38] using data collected between 1969 and 2009. Statistical analyses were carried out using PASW Statistics Release 18.0.0 (SPSS Inc., Chicago, Illinois). Bacterial concentrations, flow rate, and TSS were log_10_-transformed prior to statistical analysis to achieve normality. Analyses of variance (ANOVA) were used to test for differences between groups. All reported means are geometric means. Linear regressions were used in curve fitting and Pearson's correlations (r_p_) were calculated to characterize associations between data. Fisher's exact test was used to test for differences in the occurrence of the source tracking markers. Results with p<0.05 were deemed statistically significant.

## Results

### Flow and weather conditions

Daily average river discharge rates during our study were 3.0, 2.6, 4.5, and 5.6 m^3^/s during RC1, RC2, RC3, and RC4. The baseflow of the Hanalei River is 4.1 m^3^/s. Thus, the flow rates in 2008 were between 63% and 73% of the baseflow and in 2009 were between 110% and 137% of baseflow. There was no rainfall 5 days prior to or during the field studies.

### Historical data

Historical data considered as storm flow, non-storm-flow and combined flows were used to explore relationships between water quality parameters for the site near the Hanalei River mouth. Storm flows were defined as those greater than the 90th percentile of all historical data (10.5 m^3^/s) [29]. The mean flow rates and TSS, ENT and CP concentrations for each of the flow conditions are given in Table 3.

**Table 3:** Mean historical storm, non-storm and combined flow rates, and TSS and bacterial concentrations. Combined flows consider the entire historical record. CFU is colony forming units. MPN is most probable number.

	Storm Flow	Non-Storm Flows	Combined Flows (All)
N	27	197	224
Flow Rate (*m*^3^/s)	19.8	3.9	4.1
TSS (g/L)	3.8 × 10^−2^	4.1 × 10^−3^	5.3 × 10^−3^
ENT (MPN/100mL)	526	97	112
CP (CFU/100mL)	25	14	15

TSS, ENT and CP concentrations were significantly higher during storm flows than non-storm flows (ANOVA, p<0.001 for TSS and ENT, p<0.05 for CP). Using all the historical data (combined storm and non-storm flows), TSS is strongly, positively correlated to river flow rate r_p_ = 0.86, p < 0.01). Historical ENT is positively correlated to river flow rate (r_p_ = 0.40, p < 0.01) and TSS (r_p_ = 0.38, p < 0.01). CP also was significantly correlated to river flow rate (r_p_ = 0.16, p < 0.05) and TSS (r_p_ = 0.16, p < 0.05).

### River cruises

**Fig 3:**
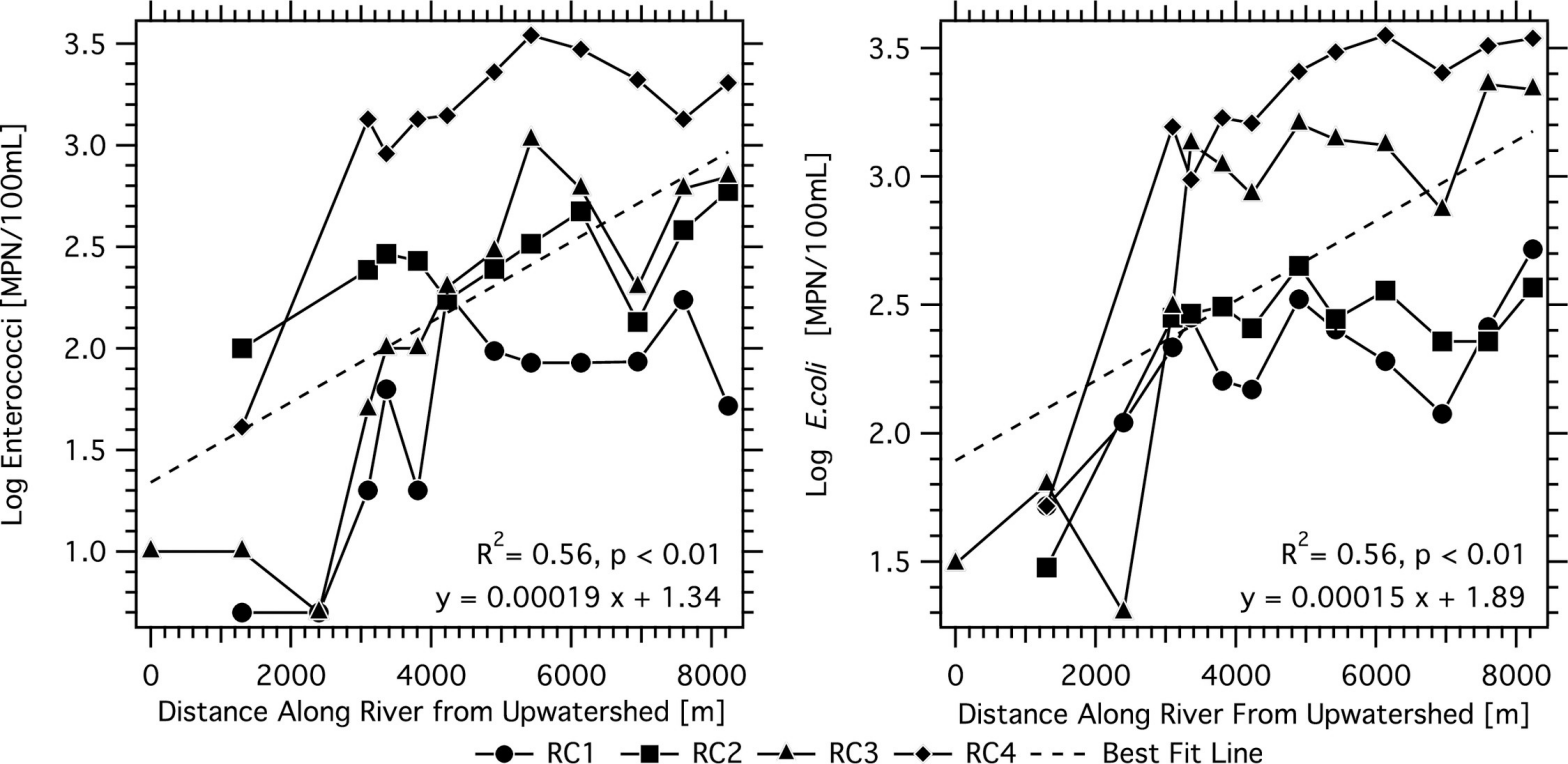
Log_10_ mean concentrations of enterococci and *E*. *coli* in surface water as function of distance along the length of the Hanalei River from the river mouth. Dashed line represents linear regression of all water samples. The distance represents the along river distance from the USGS gauge.

EC and ENT in surface water along the length of Hanalei river are shown in Fig 3 for each river cruise. Concentrations between 10 and 3470 MPN/100 mL ENT and between 20 and 3540 MPN/100 mL EC were measured; 29 of 47 measurements were above the State of Hawaii single sample standard for ENT (>104 MPN/100 mL). The concentrations of EC and ENT in the water column generally increased moving from the undeveloped upwatershed reach, through the more developed portion of the watershed, to the river mouth. A linear regression between log-transformed concentration and along river distance, from upwatershed to the river mouth, suggests an increase of 0.2 log units EC per km (r_p_ = 0.56, p < 0.01, n = 47) and 0.1 log units ENT per km (r_p_ = 0.56, p < 0.01, n = 47). ENT and EC concentrations in river water were positively correlated to each other (r_p_ = 0.84, p < 0.01).

**Fig 4:**
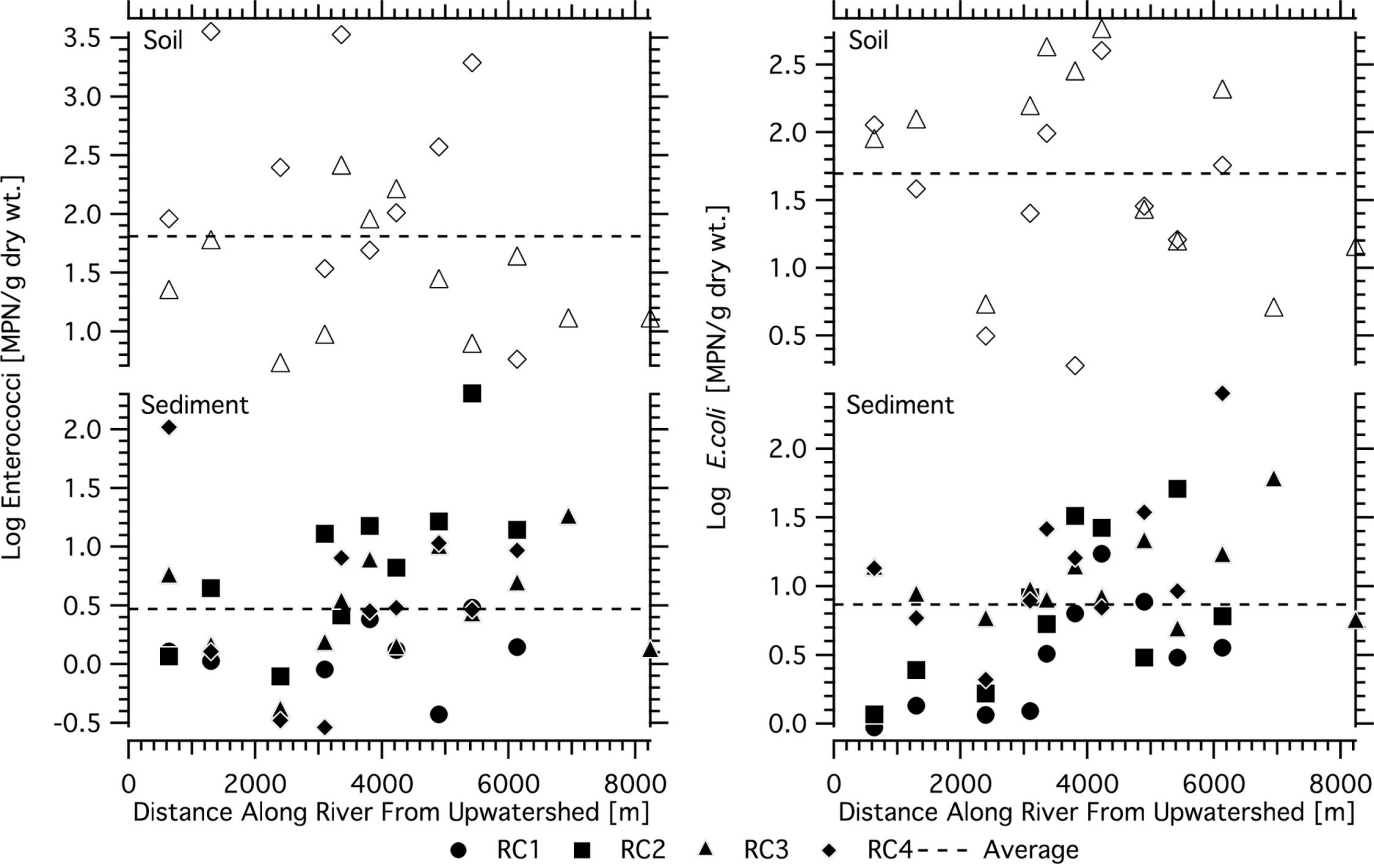
Spatial distribution of enterococci and *E*. *coli* in river sediments and soils of Hanalei River as a function of distance along the river from the river mouth. Soil concentrations are shown in top panels (squares) and sediments concentrations are shown in bottom panels (circles). Horizontal dashed lines represent average concentrations in their respective matrices. The distance represents the along river distance from the USGS gauge.

EC and ENT were detected in sediments and river bank soils along the length of the river (Fig 4). Sediment ENT ranged from 0.3 to 201.7 MPN/g while river bank soil ENT ranged from 1.8 to 3560.0 MPN/g. Sediment EC ranged from 0.9 to 252.2 MPN/g and river bank soil EC ranged from 1.9 to 554.6 MPN/g. EC and ENT generally decreased in river bottom sediments moving from upwatershed to the river mouth. However, only EC showed a significant relationship to along-river distance (r_p_ = -0.33, p<0.05), decreasing at a rate of -0.07 log units EC per km. EC and ENT concentrations in river bank soils were not correlated to along-river distance. EC and ENT concentrations were significantly higher in river bank soils compared to sediments (ANOVA, p<0.01). ENT and EC were positively correlated in sediments (r_p_ = 0.56, p < 0.01) but not in soils (r_p_ = 0.12, p = 0.56).

River water FIB, TSS, and sediment FIB concentrations at river cruise sample locations were significantly correlated. EC concentrations in water were positively correlated to TSS (r_p_ = 0.42, p < 0.01), and EC in sediments were negatively correlated to TSS (r_p_ = -0.58, p < 0.01). ENT concentrations in the water were also significantly correlated to TSS (r_p_ = = 0.44, p < 0.01), but ENT concentrations in sediments showed no significant relationship to TSS (r_p_ = -0.16, p = 0.33). FIB in soils along the river were not correlated to TSS, water FIB or sediment FIB.

River sediment types (gravel, sand, or silt) at each river cruise sampling site are shown in Table 1. All results are reported as a function of along-river distance from the most upstream sampling site (USGS gauge). Sediment type was not associated with EC or ENT in river water or sediments. EC and ENT concentrations in the river water were not significantly higher or lower when one particular sediment type was present at the base of the water column (ANOVA, p = 0.70 for both EC and ENT). Sediment type was not associated with higher or lower EC and ENT concentrations in sediment samples (ANOVA, p = 0.55 for EC and p = 0.25 for ENT).

The *Bacteroidales* fecal source tracking markers were detected in river cruise water, sediments, and soils, while the enterovirus marker was only detected in water (Table 4). Twelve of 38 river cruise water samples tested positive for at least one marker, while 6 tested positive for 2 or more markers. In the water samples, the PF marker was detected at the highest frequency (29%), the EV and CF markers were both detected in 10.5% of the samples, and the HF marker was detected least frequently in 8% of samples. ENT and EC in water samples were not significantly different in samples that tested positive versus negative for any of the molecular markers (ANOVA, p>0.34 for both EC and ENT). TSS was significantly higher in samples that were positive for the CF, and EV markers by between 1 and 1.5 mg/L (p<0.05); these differences were only weakly significant for CF and EV markers (p = 0.078 and p = 0.096, respectively).

**Table 4:** Occurrence of *Bacteroidales* host-specific fecal markers and enteroviruses in Hanalei river and potential source water, sediments and soils. Total *n* are shown in parentheses.

		HF183	CF193	PF163	EV
Hanalei River	Water Sediments	8% (38)	11% (38)	29% (38)	11%(38)
		21% (39)	0% (42)	15% (34)	0% (22)
	Soils	14% (22)	0%(22)	9% (22)	0%(22)
Ditch Inputs	Water Sediments	0% (12)	8% (12)	17% (12)	17%(12)
		56% (9)	0% (9)	11% (9)	0%(9)
Taro Field and Bird Ponds	Water Sediments	20% (5)	0% (5)	20% (5)	0%(5)
		40% (5)	0% (5)	0% (5)	0%(5)

In sediment and soil samples, only the HF and PF markers were detected. Depending on the marker, between 22 and 42 river sediment samples were assayed. No sediment samples were positive for the CF or EV markers, while 21% and 15% of the assayed river sediment samples were positive for the HF and PF markers, respectively. The occurrence of host-specific markers was assayed in 22 river bank soil samples, and the HF and PF markers were detected in 14% and 9% of the samples, respectively. ENT and EC concentrations in sediments and soils were not significantly different in samples that tested positive versus negative for any of the molecular markers (ANOVA, p>0.54 for all comparisons).

### Potential sources

The potential for ditch inputs, taro fields, and bird ponds to be sources of EC and ENT to the Hanalei river was investigated. Mean concentrations of EC and ENT in water and sediment in these samples are presented in Table 5. No significant difference was observed between FIB concentrations in ditch water compared to FIB concentrations in the Hanalei River during river cruises (ANOVA, p = 0.40 for ENT and p = 0.52 for EC). Similarly, taro lo'i water FIB concentrations were not significantly different from river water concentrations measured during river cruises (ANOVA, p = 0.20 for both EC and ENT). In bird refuge pond water, both EC and ENT were significantly higher compared to those measured in the Hanalei River by 1.7 (EC) and 1.6 (ENT) log units (ANOVA, p<0.001 for both EC and ENT). Ditch sediments had significantly higher concentrations of FIB compared to river sediments (ANOVA, p<0.05 for both EC and ENT). EC and ENT sediment concentrations in taro lo'i and bird refuge ponds were also significantly higher than Hanalei river sediments (ANOVA, p<0.001 for all comparisons), but were not significantly different from the sediment concentrations measured in the ditches.

**Table 5:** Concentration of EC and ENT in potential source water and sediment samples. Units of the reported concentration in water are MPN/100 mL and in sediments are MPN/g. The sample sizes are small for some sample types.

	Urban and Taro Lo’i Ditches		Taro Lo’i		Bird Refuge Ponds	
	Water (*n* = 12)	Sediments (*n* = 9)	Water (*n* = 3)	Sediments (*n* = 3)	Water (*n* = 2)	Sediments (*n* = 2)
EC	324	51.2	35	97.9	10	47.7
ENT	123	206.6	49	105.2	41	2304

*Bacteroidales* host-specific markers were detected in ditch, taro lo'i and bird refuge pond water, while enteroviruses were only detected in ditch water (Table 4). In ditch water samples, the PF and EV markers occurred most frequently, each in 17% of samples. The HF marker was not detected in ditch water samples but was detected in 56% of ditch sediment samples. When taro lo’i and bird refuge pond samples were examined in aggregate, the HF and PF markers occurred in 20% of water samples and the HF marker was the only marker detected in sediments where it was found 40% of the time. The occurrence of the *Bacteroidales* and enterovirus markers in ditch samples (both water and sediment) was not significantly different than their occurrence in river samples (Fisher's Exact Test, p>0.05 for both). The presence or absence of host specific markers was not associated with FIB concentrations in any of the sample matrices (ANOVA, p>0.05 for all comparisons).

### Dry weather loading of FIB in hanalei river

**Fig 5:**
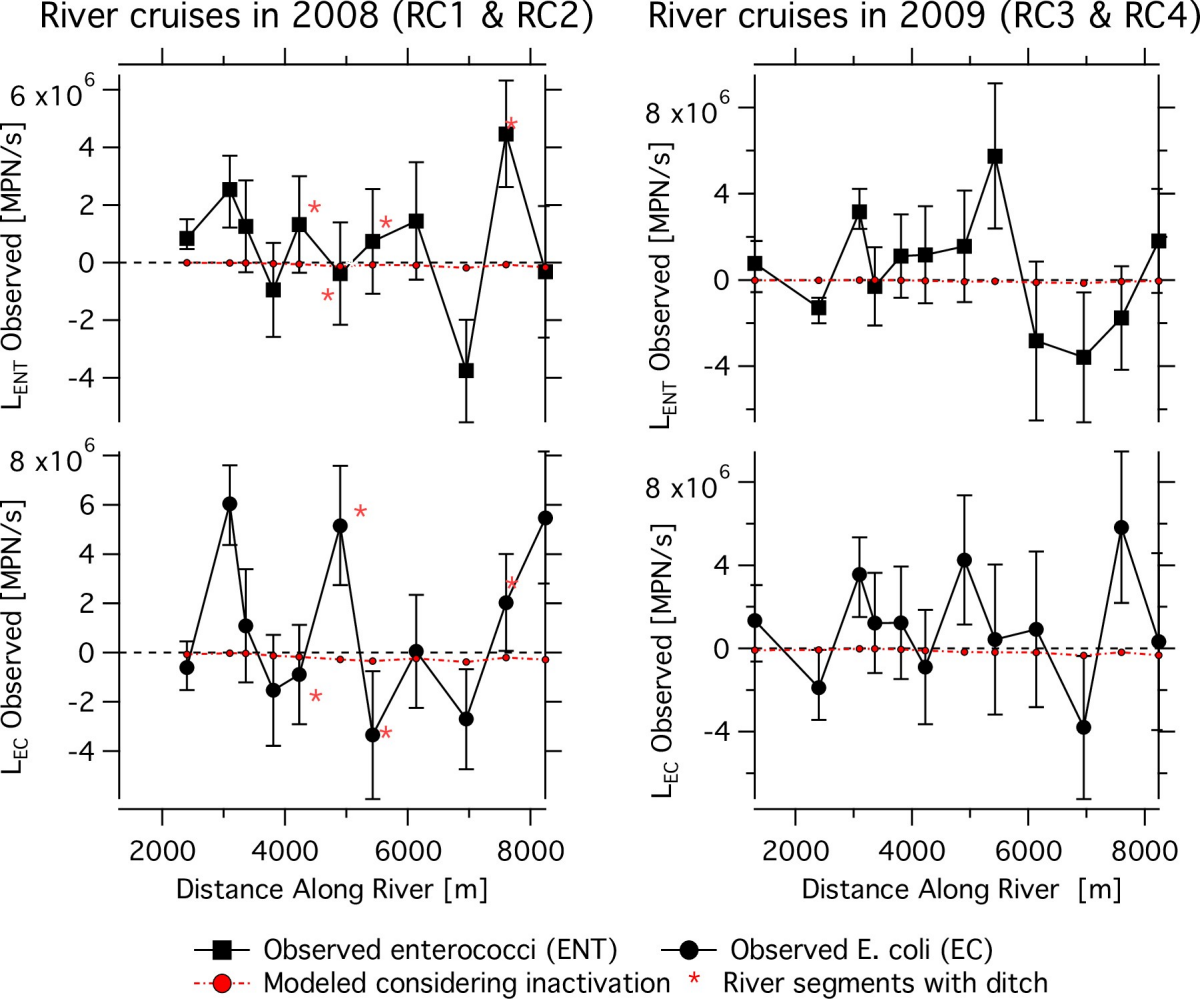
Observed and model loading results. Hanalei river loadings in 2008 (left panel) and 2009 (right panel). ENT loadings (squares) are shown in the top panels and EC loadings (circles) are shown in bottom panels. Red lines show model predictions for changes in loading due to inactivation. Red asterisks indicate river segments where a ditch drains into the river. The asterisks are only shown on the left panels. The distance represents the location of the downstream end of the river segment.

Using Eq 1, the mean observed ENT and EC load, L_obs_, in each segment of the Hanalei River was calculated for 2008 and 2009 (Fig 5). L_obs_ was different from 0 (the standard deviations did not cross 0) for only a fraction of the river segments. Only L_obs_ different from 0 are discussed. Considering both years in aggregate, the maximum observed input and loss in any river reach was 5.7 x 10^6^ MPN/s and -3.8 x 10^6^ MPN/s for ENT and 6.0 x 10^6^ MPN/s and -3.8 x 10^6^ MPN/s for EC. Distances along the river in the following paragraphs represent the end of river segments.

In 2008, observed ENT inputs occurred at 2400, 3100, and 7600 m, and losses at 6950 m; EC inputs occurred at 3100, 4900, 7600, and 8240 m and losses at 5425 and 6950 m. During 2009, ENT inputs occurred at 3100 and 5425 m, and losses at 2400 and 7600 m; EC inputs occurred at 3100, 4900 and 7600 m, and EC losses at 2400 and 6950 m. In all other river segments, loadings were not different from 0. While some river segments appear to have loading that is consistent across years and FIB (for example, inputs at 3100 m and 7600 m, and losses at 6950), other river segments appear to be sites of net input in one year and net losses in the other (for example, 2400 m).

The changing load of FIB along the river cannot be explained by deposition and resuspension of FIB-laden sediments. The change in concentration of FIB among river segments is usually on the order of 10–100 MPN/100 ml. Assuming that changes in TSS in the river reflect inputs / exports from or to the bed via resuspension and deposition, and the FIB associated with the TSS is well approximated by the concentrations we measured in the bed sediments, then the change in FIB concentrations due to resuspension and deposition are on the order of at most 10^−3^ MPN/100 ml. Therefore, it appears resuspension and deposition of sediment is not an important process controlling FIB concentrations in the river during low flow, dry weather conditions.

We investigated the potential for inactivation to explain the changes in FIB concentrations and loads along the river. Negative loading is predicted for each river segment when considering inactivation alone, and this prediction disagrees with the loadings observed in the field, which were both positive, negative and zero, depending on river segment. Even when the observed loadings were negative (indicating losses), the modeled losses were insignificant relative to the actual losses (Fig 5).

Ditches represent a potential source of FIB to the river that may explain positive loadings of FIB to the river. Ditches discharge into the river segments ending at 4230, 4900, 5425, and 7600 m (Fig 5). During 2008, significant positive loads of EC occurred in 2 of the river segments with ditches (4900 and 7600 m) and significant positive load occurred for ENT at only one of these segments with a ditch (7600 m). The same river segments (4900 and 7600 m) that had significantly positive loads in 2008 for EC, also had significant positive loads for EC in 2009. In 2009, significant positive loads of ENT occurred at a single river segment at 5425 m.

The required ditch flow rates, (Q_dit_), needed to account for the observed FIB loads were calculated for river segments that had significant positive FIB loads and contained ditches. Q_dit_, as well as 100 x Q_dit_ /Q (the percent Q_dit_ is of the Hanalei River flow rate during the RC for each year) are shown in Table 6. It appears that only the ditch draining into the river at 7600 m can explain EC and ENT loadings. Ditches draining into this segment of the river yielded required flow rates ranging from 0.09 m^3^/s to 0.15 m^3^/s, roughly 0.3% to 5.3% of the Hanalei River flow. These estimated flow rates appear to be realistic from visual river cruise observations.

**Table 6:** Required flow rates from input ditches to the Hanalei River to achieve river loadings of FIB observed in the field. Units of the reported values are m^3^/s. The corresponding percentage of the Hanalei River flow are shown in parentheses.

	2008		2009	
River Segment [m]	EC	ENT	EC	ENT
4900	2.98(107%)	*	2.46 (48.9%)	*
5425	*	*	*	4.25 (84.6%)
7600	0.01 (0.3%)	0.15 (5.3%)	0.03 (0.5%)	*

* Loads not different from 0 in Fig 5 were not evaluated.

Groundwater loadings, S, of FIB for river segments with significant positive observed FIB loads are shown in Table 7. Using Eq 4, groundwater EC loadings of 3.1 x 10^3^ MPN/s/m to 8.6 x 10^3^ MPN/s/m in 2008 and 5.1 x 10^3^ MPN/s/m to 9.0 x 10^3^ MPN/s/m in 2009 were required to account for the observed EC positive loads. Similarly, river segments with significant positive ENT loadings required groundwater ENT inputs to the river ranging from 7.7 x 10^2^ MPN/s/m to 6.9 x 10^3^ MPN/s/m in 2008 and 4.5 x 10^3^ MPN/s/m to 1.1 x 10^4^ MPN/s/m in 2009.

**Table 7:** Required groundwater fluxes of FIB to achieve river loadings of FIB observed in the field. Units of the reported values are MPN/s/m.

	2008		2009	
River Segment [m]	EC	ENT	EC	ENT
2400	*	770	*	*
3100	8.6 × 10^3^	3.6 × 10^3^	5.1 × 10^3^	4.5× 10^3^
4900	7.7 × 10^3^	*	6.4 × 10^3^	*
5425	*	*	*	1.1 × 10^4^
7600	3.1 × 10^3^	6.9 × 10^3^	9.0 × 10^3^	*
8240	8.6 × 10^3^	*	*	*

* Loads within river segments not different from 0 or negative in Fig 5 were not evaluated.

## Discussion

Soils and sediments represent sources of FIB, and human- and pig-specific fecal source tracking markers to the Hanalei River. The presence of FIB in Hawaiian soils has been documented by other researchers [10,39–41], however, this is one of the few studies to document the presence of the suite of host-associated *Bacteroidales* 16S rRNA markers in sediments and soils. When all the data are aggregated, the HF marker was 4 times as likely to be found in sediments and soil samples than water samples. Similarly, the PF marker was twice as likely to be detected in sediments or soils than in water. These data suggest that the bacteria containing these markers may have an affinity for sediment. *Bacteroides* spp. have been shown to colonize surfaces and form biofilms [42–44] that may enable them to survive and persist longer in sediments than in the water column. Interestingly, the CF and EV markers were not detected in sediments or soils. This may imply that the specific organisms containing these markers do not have an affinity for sediments and soils, or that the method used to elute bacteria and virus may not have been effective at eluting CF and EV. Previous work on EV in sewage-amended soils used beef extract to elute viruses [45,46]. It is also plausible that the CF marker and EV are not prevalent in the Hanalei watershed, personal observations by our team indicate limited cattle and goats in the watershed at the time of sampling. Further work is needed to fully understand the interaction between the host-specific markers and particles.

While sediments may be important FIB sources during storms, there is little evidence of their importance as sources during low-flow, non-storm conditions. According to the river cruise data, EC and ENT inputs and losses within the river could not be explained by deposition and resuspension of sediments. FIB concentrations would need to be at least 1000 times higher in sediments for resuspension and deposition to account for observed inputs or losses in the river segments, an unlikely possibility even considering the potential for spatial heterogeneity in the sediments. Further research should investigate additional sources of FIB to the river such as subsurface inputs.

The correlations between FIB and TSS in the water during the river cruise indicates that FIB may be particle-associated. This supports the findings of other researchers [47,48] that show strong correlations between turbidity and FIB in surface waters. Additionally, the CF and EV markers were higher when TSS was high, suggesting these markers could be associated with particles in the water column. Given these findings, and those that suggest that resuspension and deposition are not important processes affecting FIB fate during dry weather, it is likely that the organisms are associated with fine particles that remain suspended in the river water as it is transported from the mountains to the sea. Knowledge about the partitioning of FIB between planktonic and particle-associated states, and determining median diameter of particles with attached FIB will aid in understanding suspension rates of FIB within the Hanalei River [49]. Further research should investigate the association of FIB with particles of various class sizes and sedimentation rates of these particles in the Hanalei River.

Some ditches can explain increases of FIB along the river during dry weather. In particular, the ditches (D4, D5, D6 and D7 in Fig 1) discharging to the river segment ending at 7600 m can account for the increases in ENT and EC observed there during 2008 and 2009. The other ditches were either not located along segments of the river where significant FIB increases occurred, or did not have high enough concentrations to account for the observed increases in FIB. It is important to note, however, that all the ditches did contain FIB either in water or sediment, so at various times of year, they could potentially represent sources of FIB to the river. Sediments, soils, and water in taro lo'i and bird ponds contained FIB, so these can potentially seed ditch discharge with FIB.

During dry weather conditions, several river segments (2400 m, 3100 m, 4900 m and 8240 m in 2008; 3100 m, 4900 m, and 5425 m in 2009) had significant inputs of FIB that could not be explained by inputs from the ditches. The river segment at 3100 m consistently had FIB inputs and was located adjacent to agricultural lands (site CN in Fig 1), yet had no ditch input. At these locations, groundwater may represent a possible source of FIB to the river. Because the surrounding community utilizes on-site wastewater treatment systems, including cesspools, the groundwater in this area contains FIB, and previous work has identified FIB in the groundwater in some regions of Hanalei [50]. Additionally, it is known that groundwater in this region likely discharges to the river and adjacent wetlands [29]. If we use a high estimate for the concentration of FIB in groundwater, as might be expected adjacent to a cesspool of 1000 CFU/100 ml, the required flux of groundwater in river segments would range from 5 L/min/m to 50 L/min/m for both the 2008 and 2009 river cruises where the per meter refers to the length along the river. There are no estimates to our knowledge of groundwater fluxes into the Hanalei river with which to compare these estimates. The presence of the human-specific *Bacteroidales* marker in the river water, sediments and adjacent soils as well as the presence of the human enterovirus marker in the water further suggests there is wide-spread human fecal contamination in the that is likely a result of nearby wastewater disposal systems.

There were several river segments where FIB losses were observed and could not be explained by deposition of FIB to the sediments or inactivation (5425 m and 6950 m in 2008, and 2400 m and 7600 m in 2009). Other possible losses not considered in this study include removal by grazers including benthic filter feeders, as well as losses to groundwater. Future work would need to further characterize these losses in the river.

The occurrence of host-specific source tracking markers is indicative that a portion of FIB present in the Hanalei River are of fecal origin. The human-specific source tracking markers, *Bacteroidales* and enterovirus, were detected frequently in our study, suggesting human fecal inputs from septage, cesspools, or other wastewater disposal systems. The pig-specific fecal marker was also detected frequently, pointing to inputs of porcine feces in the river and watershed. This is not surprising because of the large number of feral pigs that live in the Hanalei River watershed; an animal count is not available. The ruminant-specific marker was found least often. Its presence suggests that some ruminants contribute feces to the river. Feral goats, as well as domesticated livestock, are possible sources. There is limited work on the differential persistence of these fecal markers in the environment, and more work on that may provide additional insight into their occurrence as observed in this study.

There are several limitations to the work presented herein. For the mass balance calculations we carried out, we assumed that water in the river was well mixed vertically and laterally, and that longitudinal dispersion was negligible. If there is vertical or lateral heterogeneity in FIB and TSS in the river, then sophisticated modeling would be needed to predict how various processes would affect FIB and TSS concentrations. We also assumed that the volumetric flow of water was constant as it flowed through the study site; we believe this assumption is valid, but there were small ditch inputs and potentially some water withdrawals from the river by local residence that are un-metered. Additionally, we did not attempt to determine if FIB were associated with particular size classes of particles in the study nor did we differentiate between planktonic and particle-associated bacteria in the model. The study of bacterial interaction with particles is important and is being undertaken in some laboratories [51,52] but further work is needed.

This work emphasizes the need to incorporate mass balance considerations and microbial source tracking to pin-point sources of microbial pollutant to water bodies. If the mass balance approach had not been included in the present study, river sediments and soil would be considered the probable sources of microbial contaminants to the Hanalei River during dry weather conditions based on MST results alone. However, river sediments and soils are not considered major contributors of FIB to the Hanalei River during the dry season. Instead, the modeling indicates that ditches and groundwater likely contribute substantial FIB to the river during the dry season.

## Supporting information

S1 Data (XLSX)

## References

[1] USEPA (United States Environmental Protection Agency). National Summary of State Information in the Assessment and Total Maximum Daily Load Tracking and Implementation System (ATTAINS). 2019.

[2] Ashbolt NJ, Grabow WOK, Snozzi M. Risk assessment and management for water-related infectious disease: Indicators of microbial water quality In: Fewtrell L, Bartram J, editors. Water Quality: Guidelines, Standards and Health. London: IWA Publishing; 2001 pp. 289–315.

[3] Harwood VJ, Whitlock J, Withington V. Classification of antibiotic resistance patterns of indicator bacteria by discriminant analysis: use in predicting the source of fecal contamination in subtropical waters. Applied and Environmental Microbiology. 2000;66: 3698–3704. 10.1128/aem.66.9.3698-3704.2000 10966379 PMC92209

[4] Layton BA, Walters SP, Lam L, Boehm AB. Enterococcus species distribution among human and animal hosts using multiplex PCR. Journal of Applied Microbiology. 2010;109: 539–547. 10.1111/j.1365-2672.2010.04675.x 20132375

[5] Parveen S, Portier KM, Robinson K, Edmiston L, Tamplin ML. Discriminant analysis of ribotype profiles of Escherichia coli for differentiating human and nonhuman sources of fecal pollution. Applied and Environmental Microbiology. 1999;65: 3142–3147. 10388715 10.1128/aem.65.7.3142-3147.1999 PMC91468

[6] Hardina CM, Fujioka RS. Soil: the environmental source of Escherichia coli and enterococci in Hawai‘i’s streams. Environmental Toxicology and Water Quality: An International Journal. 1991;6: 185–195.

[7] Yamahara KM, Layton BA, Santoro AE, Boehm AB. Beach sands along the California coast are diffuse sources of fecal bacteria to coastal waters. Environmental Science & Technology. 2007;41: 4515–4521. 17695890 10.1021/es062822n

[8] Soller JA, Schoen ME, Bartrand T, Ravenscroft JE, Ashbolt NJ. Estimated human health risks from exposure to recreational waters impacted by human and non-human sources of faecal contamination. Water Research. 2010;44: 4674–4691. 10.1016/j.watres.2010.06.049 20656314

[9] Fujioka RS, Tenno K, Kansako S. Naturally-Occurring Fecal Coliforms and Fecal Streptococci in Hawaiis Fresh-Water Streams. Toxicity Assessment. 1988;3: 613–630.

[10] Byappanahalli MN, Fujioka RS. Evidence that tropical soil environment can support the growth of Escherichia coli. Water Science and Technology. 1998;38: 171–174.

[11] Cui H, Yang K, Pagaling E, Yan T. Spatial and temporal variations of enterococci abundance and its relationship with microbial community in Hawai’i beach sand and water. Applied and Environmental Microbiology. 2013;79: 3601–3609. 10.1128/AEM.00135-13 23563940 PMC3675952

[12] Boehm AB, Fuhrman JA, Mrse RD, Grant SB. Tiered approach for identification of a human fecal pollution source at a recreational beach: case study at Avalon Bay, Catalina Island, California. Environmental Science & Technology. 2003;37: 673–680. 12636264 10.1021/es025934x

[13] Santoro AE, Boehm AB. Frequent occurrence of the human-specific Bacteroides fecal marker at an open coast marine beach: Relationship to waves, tides, and traditional indicators. Environmental Microbiology. 2007;9: 2038–2049. 10.1111/j.1462-2920.2007.01319.x 17635548

[14] Rosario K, Symonds EM, Sinigalliano C, Stewart J, Breitbart M. Pepper mild mottle virus as an indicator of fecal pollution. Applied and Environmental Microbiology. 2009;75: 7261–7267. 10.1128/AEM.00410-09 19767474 PMC2786529

[15] Shanks OC, Nietch C, Simonich M, Younger M, Reynolds D, Field KG. Basin-wide analysis of the dynamics of fecal contamination and fecal source identification in Tillamook Bay, Oregon. Applied and Environmental Microbiology. 2006;72: 5537–5546. 10.1128/AEM.03059-05 16885307 PMC1538696

[16] Viau EJ, Goodwin KD, Yamahara KM, Layton BA, Sassoubre LM, Burns S, et al Human bacterial pathogens and fecal indicators in tropical streams discharging to Hawaiian coastal waters. Water Research. 2011;45: 3279–3290. 10.1016/j.watres.2011.03.033 21492899

[17] Boehm AB, Yamahara KM, Walters SP, Layton BA, Keymer DP, Strickfaden RM, et al Dissolved inorganic nitrogen, soluble reactive phosphorous, and microbial pollutant loading from tropical rural watersheds in Hawai’i to the coastal ocean during non-storm conditions. Estuaries and Coasts. 2011;34: 925–936.

[18] Gawler AH, Beecher JE, Brandao J, Carroll NM, Falcao L, Gourmelon M, et al Validation of host-specific Bacteriodales 16S rRNA genes as markers to determine the origin of faecal pollution in Atlantic Rim countries of the European Union. Water Research. 2007;41: 3780–3784. 10.1016/j.watres.2007.01.028 17346765

[19] Griffith GF, Weisberg SB, McGee CD. Evaluation of microbial source tracking methods using mixed fecal sources in aqueous test samples. Journal of Water and Health. 2003; 141–151. 15382720

[20] Boehm AB, Van De Werfhorst LC, Griffith JF, Holden PA, Jay JA, Shanks OC, et al Performance of forty-three microbial source tracking methods: A twenty-seven lab evaluation study. Water Research. 2013;47: 6812–6828. 10.1016/j.watres.2012.12.046 23880218

[21] Bernhard AE, Field KG. A PCR assay to discriminate human and ruminant feces on the basis of host differences in Bacteroides-Prevotella genes encoding 16S rRNA. Applied and Environmental Microbiology. 2000;66: 4571–4574. 10.1128/aem.66.10.4571-4574.2000 11010920 PMC92346

[22] Shanks OC, White K, Kelty CA, Hayes S, Sivaganesan M, Jenkins M, et al Performance assessment PCR-based assays targeting Bacteroidales genetic markers of bovine fecal pollution. Applied and Environmental Microbiology. 2010;76: 1359–1366. 10.1128/AEM.02033-09 20061457 PMC2832389

[23] Dick LK, Bernhard AE, Brodeur TJ, Domingo JWS, Simpson J. M., Walters SP, et al Host distributions of uncultivated fecal Bacteroides bacteria reveal genetic markers for fecal source identification. Applied and Environmental Microbiology. 2005;71: 3184–3191. 10.1128/AEM.71.6.3184-3191.2005 15933020 PMC1151806

[24] Kildare BJ, Leutenegger CM, McSwain BS, Bambic DG, Rajal VB, Wuertz S. 16S rRNA-based assays for quantitative detection of universal, human-, cow-, and dog-specific fecal Bacteroidales: A Bayesian approach. Water Research. 2007;41: 3701–3715. 10.1016/j.watres.2007.06.037 17644149

[25] Noble RT, Fuhrman JD. Enteroviruses detected by reverse transcriptase polymerase chain reaction from the coastal waters of Santa Monica Bay, California: low correlation to bacterial indicator levels. Hydrobiologia. 2001;460: 175–183.

[26] Mocé-Llivina L, Lucena F, Jofre J. Enteroviruses and bacteriophages in bathing waters. Applied and Environmental Microbiology. 2005;71: 6838–6844. 10.1128/AEM.71.11.6838-6844.2005 16269717 PMC1287676

[27] Boehm AB, Yamahara KM, Love DC, Peterson BM, McNeill K, Nelson KL. Covariation and photoinactivation of traditional and novel indicator organisms and human viruses at a sewage-impacted marine beach. Environmental Science & Technology. 2009;43: 8046–8052. 19924921 10.1021/es9015124

[28] Boehm AB, Griffith J, McGee C, Edge TA, Solo-Gabriele HM, Whitman R, et al Faecal indicator bacteria enumeration in beach sand: a comparison study of extraction methods in medium to coarse sands. Journal of applied microbiology. 2009;107: 1740–50. 10.1111/j.1365-2672.2009.04440.x 19659700 PMC2810257

[29] TetraTech Inc., State of Hawaii Department of Health. EPA National Summary of Impaired Waters and TMDL Information—TMDL ID: 35294. 2010. Available: http://oaspub.epa.gov/tmdl/waters_list.tmdl_report?p_tmdl_id=35294

[30] Walters SP, Field KG. Persistence and growth of fecal Bacteroidales assessed by bromodeoxyuridine immunocapture. Applied and Environmental Microbiology. 2006;72: 4532–9. 10.1128/AEM.00038-06 16820440 PMC1489324

[31] Walters SP, Field KG. Survival and persistence of human and ruminant-specfiic fecal Bacteroidales in freshwater microcosms. Environmental Microbiology. 2009;11: 1410–1421. 10.1111/j.1462-2920.2009.01868.x 19397677

[32] Fuhrman JA, Liang X, Noble RT. Rapid detection of enteroviruses in small volumes of natural waters by real-time quantitative reverse transcriptase PCR. Applied and Environmental Microbiology. 2005;71: 4523–4530. 10.1128/AEM.71.8.4523-4530.2005 16085845 PMC1183282

[33] Walters SP, Yamahara KM, Boehm AB. Persistence of nucleic acid markers of health-relevant organisms in seawater microcosms: Implications for their use in assessing risk in recreational waters. Water Research. 2009;43: 4929–4939. 10.1016/j.watres.2009.05.047 19616273

[34] Hurley MA, Roscoe ME. Automated statistical analysis of microbial enumeration by dilution series. Journal of Applied Bacteriology. 1983;55: 159–164.

[35] Boehm AB, Keymer DP, Shellenbarger GG. An analytical model of enterococci inactivation, grazing, and transport in the surf zone of a marine beach. Water Research. 2005;39: 3565–3578. 10.1016/j.watres.2005.06.026 16095656

[36] Sinton LW, Hall CH, Lynch PA, Davies-Colley RJ. Sunlight inactivation of fecal indicator bacteria and bacteriophages from waste stabilization pond effluent in fresh and saline waters. Applied and Environmental Microbiology. 2002;68: 1122–1131. 10.1128/AEM.68.3.1122-1131.2002 11872459 PMC123754

[37] Gustard A, Bullock A, Dixon JM. Low flow estimation in the United Kingdom. Institute of Hydrology; 1992. Report No.: 108.

[38] Jordan TE, Correll DL, Weller DE. Relating nutrient discharges from watersheds to land use and streamflow variability. Water Resources Research. 1997;33: 2579–2590.

[39] Byappanahalli M, Fujioka R. Indigenous soil bacteria and low moisture may limit but allow faecal bacteria to multiply and become a minor population in tropical soils. Water Science and Technology. 2004;50: 27–32. 15318482

[40] Roll BM, Fujioka RS. Sources of faecal indicator bacteria in a brackish, tropical stream and their impact on recreational water quality. Water Science and Technology. 1997;35: 179–186.

[41] Oshiro R, Fujioka R. Sand, soil, and pigeon droppings: Sources of indicator bacteria in the waters of Hanauma Bay, Oahu, Hawaii. Water Science and Technology. 1995;31: 251–254.

[42] Olapade OA, Depas MM, Jensen ET, McLellan SL. Microbial communities and fecal indicator bacteria associated with Cladophora mats on beach sites along Lake Michigan shores. Applied and Environmental Microbiology. 2006;72: 1932–1938. 10.1128/AEM.72.3.1932-1938.2006 16517640 PMC1393218

[43] Macfarlane S, Macfarlane GT. Composition and metabolic activities of bacterial biofilms colonizing food residues in the human gut. Applied and Environmental Microbiology. 2006;72: 6204–6211. 10.1128/AEM.00754-06 16957247 PMC1563644

[44] Probert HM, Gibson GR. Bacterial biofilms in the human gastrointestinal tract. Current Issues In Intestinal Microbiology. 2002;3: 23–7. 12400635

[45] Landry EF, Vaughn JM, Thomas MZ, Beckwith CA. Adsorption of enteroviruses to soil cores and their subsequent elution by artificial rainwater. Appl Environ Microbiol. 1979;38: 680–7. 231936 10.1128/aem.38.4.680-687.1979 PMC243560

[46] Straub TM, Pepper IL, Abbaszadegan M, Gerba CP. A method to detect enteroviruses in sewage sludge-amended soil using the PCR. Applied and Environmental Microbiology. 1994;60: 1014–7. 8161168 10.1128/aem.60.3.1014-1017.1994 PMC201425

[47] Surbeck CQ, Jiang SC, Ahn JH, Grant SB. Flow Fingerprinting Fecal Pollution and Suspended Solids in Stormwater Runoff from an Urban Coastal Watershed. Environmental Science and Technology. 2006;40: 4435–4441. 10.1021/es060701h 16903282

[48] Huey GM, Meyer ML. Turbidity as an Indicator of Water Quality in Diverse Watersheds of the Upper Pecos River Basin. Water. 2010;2: 273–284. 10.3390/w2020273

[49] Bradford SA, Morales VL, Zhang W, Harvey RW, Packman AI, Mohanram A, et al Transport and Fate of Microbial Pathogens in Agricultural Settings. Critical Reviews in Environmental Science and Technology. 2013;43: 775–893. 10.1080/10643389.2012.710449

[50] Knee KL, Layton BA, Street J, Boehm AB, Paytan A. Sources of nutrients and fecal indicator bacteria to nearshore waters on the North Shore of Kaua`i. Estuaries and Coasts. 2008;31: 607–622.

[51] Fries JS, Characklis GW, Noble RT. Attachment of Fecal Indicator Bacteria to Particles in the Neuse River Estuary, N.C. Journal of Environmental Engineering. 2006;132: 1338–1345.

[52] Characklis GW, Dilts MJ, Simmons OD, Likirdopulos CA, Krometis LAH, Sobsey MD. Microbial partitioning to settleable particles in stormwater. Water Research. 2005;39: 1773–1782. 10.1016/j.watres.2005.03.004 15899275

